# Plasma renin as a novel prognostic biomarker of sepsis-associated acute respiratory distress syndrome

**DOI:** 10.1038/s41598-024-56994-3

**Published:** 2024-03-20

**Authors:** Anjali Chakradhar, Rebecca M. Baron, Mayra Pinilla Vera, Prasad Devarajan, Lakhmir Chawla, Peter C. Hou

**Affiliations:** 1 Department of Molecular and Cellular Biology, Harvard University, Cambridge, MA USA; 2 Division of Pulmonary Critical Care Medicine, Department of Medicine, Brigham and Women’s Hospital, Boston, MA USA; 3 Department of Pediatrics, University of Cincinnati College of Medicine, Cincinnati, OH USA; 4 Silver Creek Pharmaceuticals, Inc., San Francisco, CA USA; 5 Division of Emergency Critical Care Medicine, Department of Emergency Medicine, Brigham and Women’s Hospital, Boston, MA USA

**Keywords:** Renin, Sepsis, Acute respiratory distress syndrome (ARDS), Septic shock, Mortality, Critical care, Respiratory signs and symptoms, Prognostic markers

## Abstract

Sepsis-associated acute respiratory distress syndrome (ARDS) is a life-threatening condition in critical care medicine for which there is a substantial need for early prognostic biomarkers of outcome. The present study seeks to link plasma renin levels and 30-day mortality in sepsis-associated ARDS patients treated at our institution. The Registry of Critical Illness (RoCI) prospectively enrolled patients from the intensive care units (ICU) within a single academic medical center, and a convenience sample of patients with sepsis-associated ARDS was analyzed from this cohort. This study was approved by the Mass General Brigham Institutional Review Boards (IRB) as part of the RoCI, and all procedures performed were in accordance with the ethical standards of the institutional board. From April 2012 to February 2019, a cohort of 32 adult sepsis-associated ARDS patients with 500 µL of plasma samples available on Day 0 and Day 3 of their ICU stay were enrolled. Renin levels were measured twice, on Day 0 and Day 3 via the direct renin enzyme-linked immunosorbent assay (ELISA EIA-525) by DRG diagnostics. Day 0 and Day 3 renin were statistically evaluated via logistic regression to predict 30-day mortality. Direct renin levels of 64 samples were assayed from 32 sepsis-associated ARDS patients (50% male; mean ± SD, 55 ± 13.8 years old). The 30-day hospital mortality rate was 59.4%. Patients who died within 30 days of admission were more likely to have an elevated Day 3 Renin (Odds ratio [OR] = 6, 95% CI 1.25–28.84) and have received vasopressors (OR = 13.33, 95% CI 1.43–123.95). Adjusting for vasopressor use as a proxy for septic shock status, patients with an Elevated Day 3 Renin had a 6.85 (95% CI 1.07–43.75) greater odds of death than those with Low-Normal Day 3 Renin. Patients with sustained Elevated Renin levels from Day 0 to Day 3 had the highest risk of death in a 30-day window. In this study, we found that renin may be a novel biomarker that has prognostic value for patients with sepsis-associated ARDS. Future studies evaluating renin levels in patients with sepsis-associated ARDS are needed to validate these findings.

Sepsis is the leading cause of death in US hospitals and a common cause of acute respiratory distress syndrome (ARDS). ARDS is a devastating form of lung failure with limited treatment options and mortality rate of 35–50%^1^. ARDS is also frequently seen in patients hospitalized with SARS-CoV2, making the need to develop prognostic biomarkers that are readily available for clinical use increasingly critical. Biomarkers are useful indicators whose presence and levels help guide understanding of disease progression and treatment efficacy. While some progress has been made in understanding sub-phenotypes of ARDS^2^, there still remains a substantial need for early prognostic biomarkers of outcome in this condition.

Renin has been well studied in the context of hypertension. The renin-angiotensin system (RAS) is an important circulatory homeostatic mechanism that regulates blood pressure, inflammation, and immune responses. Renin is secreted in response to decreased tissue-perfusion and hypoxia by renin-expressing cells in the kidney. Renin catalyzes the conversion of angiotensinogen to angiotensin I, which is then converted via the angiotensin converting enzyme (ACE) into angiotensin II (Ang II). Via biofeedback mechanisms, angiotensin II is a very powerful direct inhibitor of renin secretion. Sepsis-associated ARDS is characterized by pulmonary endothelial dysfunction and is associated with decreased serum ACE^4^. Decreased serum ACE levels result in decreased Ang II level and since Ang II is a powerful direct inhibitor of renin secretion, decreased Ang II level results in increased renin levels. Hence, elevated plasma renin may indicate more severe pulmonary capillary endothelial dysfunction and may be an important surrogate of worsening disease condition.

Prior literature suggests that RAS plays a role in the pathogenesis of non-COVID ARDS^3,4,10,11^, with particularly increased recent attention to this pathway given SARS-CoV2 binding to the ACE2 receptor on lung epithelial cells, in particular^12–14^. In 2019, Gleeson et al. demonstrated that maximum renin level over the course of a patient's hospital stay achieved significant prognostic value for hospital mortality in a heterogeneous critically ill population^3^. We sought to determine if elevated plasma renin levels signaled worse outcomes in the sepsis-associated ARDS patients treated at our institution. Our study aims to assess the prognostic value of plasma renin levels over two time points in a cohort of non-COVID sepsis-associated ARDS patients as a predictor of hospital mortality. This study was done as part of an exploratory study using state of the art measurement of renin levels.

We investigated renin as a biomarker for sepsis-associated ARDS instead of ACE because renin is upstream to both ACE and ACE2^16^. Additionally, both ACE and ACE2 are involved in the AT1 and AT2 receptor pathways, which are implicated in the development and progression of sepsis-associated ARDS. Specifically, the AT1 receptor pathway is known to exacerbate ARDS, while the AT2 receptor pathway has been demonstrated as preventing or even protecting against ARDS. However, both AT1 and AT2 pathways are known to involve renin, making renin a promising target for further investigation as a potential biomarker for sepsis-associated ARDS. Furthermore, renin is the rate-limiting enzyme in the RAAS pathway, allowing changes in renin levels to occur earlier and be more sensitive indicators of RAAS activation than ACE levels.

## Methods

### Sample collection and measurements

The Registry of Critical Illness (RoCI) and its inclusion and exclusion criteria are described by Dolinay et al.^5^. Notable exclusion characteristics include subjects unable to provide consent and whose legal representatives could not be found, subjects admitted to the ICU for end-of-life comfort care, and those with a hemoglobin level of < 8 g/dL or a rare blood type. Blood draws were not done in these patients due to risks of anemia and IRB regulations by Brigham and Women’s Hospital around critically ill patients. Blood was collected and transferred in EDTA coated blood collection tubes, processed within 4 h of venipuncture, and plasma was stored at − 80 °C. Extensive data including demographics (age, sex, race), severity of illness (APACHE II Score, SOFA Score), body mass index (BMI), diabetes status, chronic obstructive pulmonary disease (COPD) status, smoking history, pneumonia status, vasopressor use, and PaO_2_/FiO_2_ were collected. For this study, we retrospectively evaluated patient data collected between April 2012 and February 2019 and identified patients who met inclusion criteria for sepsis-associated ARDS and had sufficient plasma samples (500 µL) needed for renin evaluation on Day both 0 and Day 3. The RoCI had 430 patients of whom 322 patients had sepsis only and 108 had sepsis-associated ARDS. Since several of the RoCI patients with sepsis-associated ARDS were enrolled in previous studies that consumed their available samples, 32 patients remained with an adequate quantity of blood samples to undergo renin level testing on both Day 0 and Day 3 following their admission to the ICU, and were therefore included in this study.

Aliquots of plasma were shipped to the Devarajan laboratory in the Division of Nephrology and Hypertension at Cincinnati Children’s Hospital Medical Center. Renin measurements were obtained using the specialized active renin ELISA kit from DRG Diagnostics (Kit Reference ID: EIA-5125). Renin assays were performed in November 2019. The assay dynamic range is 0.80–128.0 pg/ml. The intra-assay coefficient of variability (CV) is 2.4% and the inter-assay CV is 3.7%. The assay has a sensitivity of 0.80 pg/mL, and no cross sensitivity detected with serum albumin, gamma globulin, hepcidin, and pepsin. For the expected normal values, in a study conducted with healthy adults in the supine position, the range was 2.13–58.78 pg/ml^15^.

### Statistical analyses

Renin levels at Day 0 (D0 Renin) and Day 3 (D3 Renin) were the statistical exposures of interest and 30-day mortality was the primary outcome. As per the cutoff reported by the DRG Renin Data Sheet-5125, samples were classified as either Low-Normal Renin (< 58.78 pg/ml) and Elevated Renin (≥ 58.78 pg/ml). We used the categorical change in renin from D0 to D3 to evaluate the association between the longitudinal renin levels and 30-day mortality. Patients were split into four categories: (0) Low-Normal D0 Renin to Low-Normal D3 Renin, (1) Elevated D0 Renin to Low-Normal D3 Renin, (2) Low-Normal D0 Renin to Elevated D3 Renin, (3) Elevated D0 Renin to Elevated D3 Renin. There were 15 patients in Category (0), 5 patients in Category (1), 4 patients in Category (2), and 8 patients in Category (3). This categorical definition of ΔRenin along with the number of patients in each category can also be found in Appendix Table 1. We plotted Kaplan–Meier survival curves to visually evaluate the association between renin and 30-day hospital mortality, which can be found in Appendix Figs. 1 and 2. We conducted univariate logistic regressions to evaluate the associations between potential predictors (including renin levels) and 30-day hospital mortality. Only variables with a p < 0.1 in bivariate analyses were incorporated into the multivariate logistic regression models to evaluate the association between the renin values and 30-day hospital mortality. We also evaluated the diagnostic test accuracy of using Day 0 and Day 3 renin values to predict 30-day hospital mortality. To explore the predictive window of renin level on the hospital mortality, we conducted a sensitivity analysis to evaluate the association between renin values and 15-day hospital mortality. We also performed sensitivity analyses, considering renin as a continuous variable for 30-day hospital mortality. p < 0.1 in regression analyses were considered significant. Statistics were performed using Prism 8 (GraphPad Software, San Diego, CA) and Jupyter Notebook using Python statsmodels v0.12.0 (IPython).

### Ethics approval and consent to participate

This study was approved by the Mass General Brigham Institutional Review Boards (IRB) as part of the RoCI, and all procedures performed were in accordance with the ethical standards of the institutional board. Informed consent was obtained from all subjects and/or their legal guardian(s) and methods were in accordance with scientific guidelines and regulations.

## Results

### Patients

A cohort of 32 sepsis-associated ARDS patients was assembled from the Registry of Critical Illness (RoCI). Baseline characteristics are described in Appendix Tables 2a and 2b and stratified by 30-day hospital mortality status as shown in Table 1. The average age was 55 years (SD ± 13.8). Twenty-eight patients (87.5%) were White non-Hispanic. Mean APACHE Score was 31 (SD ± 7.1) and 65.6% of patients were on vasopressors. The 30-day hospital mortality rate 59.4%.

**Table 1:** Baseline characteristics for 30-day mortality survivors and nonsurvivors.

Characteristic	All patients No. (%), N = 32		
		30-day mortality status	
		Survivors No. (%), N = 19	Nonsurvivors No. (%), N = 13
Age *(mean*$\pm$*sd)*	55.4$\pm$13.8	53.4$\pm$15.0	58.3$\pm$11.8
Male Sex	16 (50%)	8 (42%)	8 (62%)
Race			
Black	2 (6%)	1 (5%)	1 (8%)
Hispanic	2 (6%)	2 (11%)	0 (0%)
White	28 (88%)	16 (84%)	12 (92%)
BMI *(mean*$\pm$*sd)* (N = 28)*	28.9$\pm$6.2	30.6$\pm$6.7	26.6$\pm$4.7
Diabetes	9 (28%)	7 (37%)	2 (15%)
COPD	4 (13%)	2 (11%)	2 (15%)
History of Smoking (N = 27)*	12 (44%)	8 (57%)	4 (31%)
Has Pneumonia	20 (63%)	11 (58%)	9 (69%)
APACHE II *(mean*$\pm$*sd)*	30.7$\pm$7.1	30$\pm$6.8	31.6$\pm$7.6
SOFA *(mean*$\pm$*sd)* (N = 31)*	8.8$\pm$4.5	9.1$\pm$4.1	8.4$\pm$5.2
On Vasopressors	21 (66%)	9 (47%)	12 (92%)
Day 0 PaO2/FiO2 *(mean*$\pm$*sd)*	177.2$\pm$74.4	183.8$\pm$66.4	167.4$\pm$86.8
Day 3 PaO2/FiO2 *(mean*$\pm$*sd)*	217.8$\pm$98.3	219.7$\pm$62.9	214.9$\pm$137.9
Δ PaO2/FiO2 Day 3- Day 0 *(mean*$\pm$*sd)*	40.6$\pm$96.9	35.9$\pm$85.5	47.4$\pm$114.8
Day 0 Renin pg/mL *(mean*$\pm$*sd)*	63.8$\pm$68.2	52.0$\pm$63.2	81.1$\pm$74.0
Day 3 Renin pg/mL *(mean*$\pm$*sd)*	68.2$\pm$110.2	32.5$\pm$42.5	120.3$\pm$153.9

*Sample size varied due to missing data.

### Renin levels and 30-day mortality

In the univariate analyses, patients who died within 30 days of admission to the hospital were more likely to have received vasopressors (Odds ratio [OR] = 13.33, 95% CI 1.43–123.95) as seen in Table 2, have Elevated D3 Renin (OR = 6, 95% CI 1.25–28.84), or have a sustained Elevated Renin level on both Day 0 and Day 3 (OR = 8.25, 95% CI 1.15–59) as seen in Table 3 (Appendix Figs. 1 and 2). On Day 0, 13 patients (40.6%) had Elevated Renin. On Day 3, 12 (37.5%) had Elevated Renin. After adjusting for vasopressor use, patients with an Elevated D3 Renin had a 6.85 greater odds of death (95% CI 1.07–43.75) than those with Low-Normal D3 Renin, as seen in Table 3. While comprehensive data on the duration of mechanical ventilation, duration of vasopressor support, and ventilator-free days is currently unavailable, pertinent information on renal replacement therapy has been obtained. Specifically, in the two patients on renal replacement therapy, one had a D0 Renin of 45.018 pg/mL, escalating to 69.09 pg/mL on D3, making them Category 2. The other patient had D0 Renin 131.05 pg/mL and D3 Renin at 133.08 pg/mL, making them Category 3.

**Table 2:** Univariate analysis of association between co-variates and 30-day mortality (N = 32).

Covariates	OR (95% CIs)	P-Values
Age *(continuous)*	1.03 (0.97–1.09)	0.324
Race		
Black	Ref	-
Hispanic	- †	-
White	0.75 (0.04–13.24)	0.844
Sex		
Female	Ref	-
Male	2.2 (0.52–9.3)	0.284
Has COPD		
No	Ref	-
Yes	1.55 (0.19–12.64)	0.685
With history of smoking (N = 27)*		
No	Ref	-
Yes	0.33 (0.07–1.62)	0.174
Has pneumonia		
No	Ref	-
Yes	1.64 (0.37–7.25)	0.517
Has diabetes		
No	Ref	-
Yes	0.31 (0.05–1.83)	0.197
On vasopressors		
No	Ref	-
Yes	13.33 (1.43–123.95)	0.023
BMI *(continuous)* (N = 28)*	0.88 (0.74–1.03)	0.108
APACHE *(continuous)*	1.03 (0.93–1.15)	0.521
SOFA *(continuous)* (N = 31)*	0.96 (0.82–1.13)	0.652
Day 1 PaO2/FiO2 *(continuous)*	1 (0.99–1.01)	0.536
Day 3 PaO2/FiO2 *(continuous)*	1 (0.99–1.01)	0.889
ΔPaO2/FiO2 *(continuous)*	1.01 (1–1.01)	0.213

^†^Lack of outcome.

*Sample size varied due to missing data.

Ref is shorthand for reference category used in the regression.

**Table 3:** Univariate and multivariate analyses of exposures for 30-day mortality.

	Univariate		Multivariate*	
	OR (95% CIs)	P-values	OR (95% CIs)	P-values
Day 0 Renin				
Low-normal	Ref	-	Ref	-
Elevated	2.53 (0.59–10.86)	0.212	2 (0.39–10.15)	0.405
Day 3 Renin				
Low-normal	Ref	–	Ref	–
Elevated	6 (1.25–28.84)	0.025	6.85 (1.07–43.75)	0.042
ΔRenin				
D0 Low_Normal to D3 Low_Normal	Ref	–	Ref	–
D0 Elevated to D3 Low_Normal	0.69 (0.06–8.15)	0.766	0.65 (0.05–9.15)	0.747
D0 Low_Normal to D3 Elevated	2.75 (0.28–26.61)	0.382	5.39 (0.3–97.01)	0.253
D0 Elevated to D3 Elevated	8.25 (1.15–59)	0.036	6.57 (0.74–58.32)	0.091

*Adjusted for Vasopressors.

The diagnostic test accuracy of binary renin, either Low-Normal or Elevated on specific days (D0 or D3), in relation to 30-day hospital mortality, is presented in Tables 3a and 3b of the appendix. The binary D3 renin level discriminated between patients who died within 30 days of admission and those who did not (area under the receiver operating curve [ROC AUC] = 0.702, 95% CI 0.536–0.869), which closely resembles the results obtained using continuous D3 renin level (AUC = 0.725, 95% CI 0.540–0.909). According to the ROC of the continuous D3 renin model, the optimal cutoff with a specificity of 0.8 and a sensitivity of 0.6 provided the best model discrimination (Appendix Fig. 3). The choice of the cutoff (58.78 pm/mL) for the binary D3 Renin value in our study results in a specificity of 0.79 and a sensitivity of 0.62, which is also very close to the optimal D3 cutoff (Appendix Table 3b). We also found that patients with sustained Elevated Renin levels had the highest risk of death in a 30-day window.

In the sensitivity analyses, as shown in Table 4, when employing 15-day mortality as the outcome variable, it was observed that patients with an Elevated D0 Renin exhibited 10.5 times higher odds of mortality compared to those with a Low-Normal Renin (OR = 10.5, 95% CI 1.36–81.06). In the sensitivity analyses with renin as a continuous variable, it was observed that Day 3 renin was associated with 30-day in-hospital mortality in a univariate model. However, this association did not persist after adjusting for the use of vasopressors, as indicated in Appendix Table 4.

**Table 4:** Univariate and multivariate analyses of exposures for 15-day mortality.

	Univariate		Multivariate	
	OR (95% CI)s	P-values	OR (95% CIs)	P-values
Day 0 renin				
Low-normal	Ref	–	Ref	–
Elevated	9.92 (1.6–61.60)	0.014	10.5 (1.36–81.06)	0.024
Day 3 Renin				
Low-normal	Ref	–	Ref	–
Elevated	5.67 (1.07–30.08)	0.042	6 (0.89–40.31)	0.065
ΔRenin				
D0 Low_Normal to D3 Low_Normal	Ref	–	Ref	–
D0 elevated to D3 Low_Normal	1.63 (0.11–22.98)	0.719	1.75 (0.1–30.84)	0.702
D0 Low_Normal to D3 Elevated	– ^†^	–	– ^†^	–
D0 Elevated to D3 Elevated	19.5 (2.19–173.49)	0.008	21 (1.5 -293.27)	0.024

^†^Lack of outcome.

## Discussion

**Figure 1:**
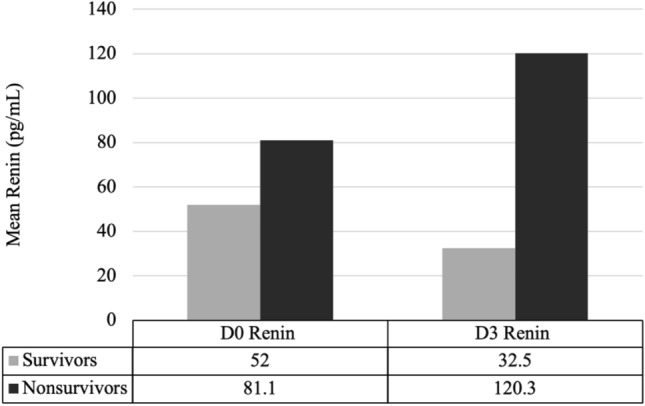
Divergence of Renin in Survivors vs Nonsurvivors from Day 0 to Day 3.

In this cohort of sepsis-associated ARDS patients, Day 3 Renin was found to be a potential biomarker that could prognosticate hospital mortality. In our study, we show that patients with an Elevated Day 3 Renin level had a more than sixfold risk of death in 30 days. We also show that patients with a sustained Elevated Renin level have greatest risk of death with an 8.25-fold greater risk compared to patients with sustained Low-Normal Renin. Renin trajectory contrasted between survivors and non-survivors as depicted in Fig. 1. These findings are novel not only because of plasma renin analysis, but also because they give evidence for the importance of patient trajectory early on in disease course. Given the smaller sample size, the power of this study is increased by looking at plasma renin at multiple time points per patient.

Our findings support the results from Gleeson and colleagues which assessed a 20-patient heterogeneous ICU cohort, and measured renin levels at 6-h intervals for up to 2 days per patient. In contrast, our study had 32 sepsis-associated ARDS patients with 2 measurements of renin levels per patient. It has been posited that renin could rival lactate’s established ability as a biomarker for mortality in critically ill patients, and further investigation of renin on the critically ill population has been urged^6^. While Gleeson (2019) examined 20 ICU patients with diverse medical conditions, this study investigated a cohort of 32 patients all with sepsis-associated ARDS. This shift in patient population is better positioned to establish a clear connection between renin and the ARDS progression in sepsis patients. Gleeson (2019) included more data points per patient but in a shorter time frame (an average of 5.5 renin measurements per patient over an average of 33 h) while this study encompassed Day 3 of ICU stays. This extended data provides a unique opportunity to look past initial fluctuations in renin levels associated with emergent interventions frequently performed during early ICU admission and elucidate the underlying relationship between renin and ARDS pathogenesis. Therefore, Day 3 renin is a powerful data point that adds a distinctive dimension to this research. Finally, Gleeson (2019) employed a pro-renin assay while this study used a direct-renin assay that was chosen for its ability to provide a more precise and reliable measurement of renin levels, as discussed further below.

To support the importance of elevated renin levels seen on ICU Day 3, Zhang et.al. found that low levels of Ang II on ICU Day 3 in severe sepsis patients was a significant factor in predicting mortality in 2014^8^. As postulated, low levels of Ang II level was due to binding of Ang II to their receptors, which can then stimulate proinflammatory and profibrotic pathways that would further worsen gas exchange in the lungs. Via biofeedback mechanisms, Ang II has been shown to be a powerful inhibitor of renin secretion. This is supported by a post-hoc of the "Angiotensin II for the Treatment of Vasodilatory Shock (ATHOS-3) trial” that found elevated renin levels in patients with catecholamine-resistant distributive shock^9^, and when Ang II was administered, serum renin levels decreased, which supports that the use of Ang II can suppress serum renin levels. In this same study, renin levels were elevated in ARDS patients as compared to non-ARDS patients^9^.

As of March 8th, 2023, a PubMed search of “Renin, Sepsis, ARDS, Mortality” yields 8 results, 4 of which were within the last year, and none of which assayed for renin level. Our study provides novel evidence that renin may be a new biomarker to prognosticate sepsis-associated ARDS mortality. These findings are consistent with a study by Chung et al. who found plasma renin activity (PRA) was a significant predictor of mortality in patients with septic shock. Our cohort measured direct plasma renin rather than PRA. As noted by Gleeson et.al., direct plasma renin is more reflective of tissue perfusion than PRA because PRA is an indirect measurement of renin and actually measures angiotensinogen, the substrate that renin catalyzes the conversion into angiotensin I. As a result, PRA is influenced by hepatic function, corticosteroids, endotoxins, and other factors that are variable in critically ill patients^3^.

An interesting point is the seemingly high observed mortality rate within our cohort of 59.4%. This may be partly because Brigham and Women’s Hospital functions as a tertiary care center, thus accommodating a more critically ill population with sepsis-associated ARDS. The average APACHE score of 30.66 supports the idea of a high-mortality cohort. Furthermore, the observed mortality rate aligns with findings reported by Dolinay (2012) in a distinct cohort from the RoCI^5^. In that study, the mortality for sepsis-associated ARDS was 60.7%, and the mean APACHE 2 score was 30. Moreover, the mortality rate for patients with sepsis-associated ARDS across the entire RoCI stands at 51.2% (60 out of 117, up to subject 718).

This study has limitations. First, a small sample of patients with 500µL at 2 time points was available. Although we found a statistically significant association, the small sample size led to wider confidence intervals. This is still a valuable finding because high quality data on sepsis-associated ARDS patients is not easily obtained. Second, Day 0 Renin level (and change in renin level) was not significantly predictive of 30-day hospital mortality; however, there may be a signal in the sensitivity analysis as a predictor of 15-day hospital mortality. Third, this study did not investigate the underlying cause of elevated renin levels, such as increased production or decreased clearance. Finally, exclusion criteria did not account for medications that could affect renin levels, which could impact results. We hypothesize that Day 3 Renin was more predictive than Day 0 by virtue of D3 Renin being later in the treatment trajectory. On day 3, patients may have reached a homeostatic state of critical illness so that renin levels can be more predictive of 30-Day mortality. A combination of pre-ICU interventions could have fluctuated Day 0 Renin levels, allowing a more stable measurement on Day 3. Despite these limitations, renin emerges as a promising biomarker for sepsis-associated ARDS. The direct renin ELISA (EIA-525) by DRG diagnostics used in this study to measure renin can be measured using a commercially available platform which can be placed in any standard clinical hospital laboratory setting. The typical turnaround time is 4–6 h, demonstrating the promise of renin levels as a bedside measurement in guiding ICU care for patients on the horizon.

## Conclusion

In a cohort of sepsis-associated ARDS patients, Renin was found to be a novel biomarker with the potential to prognosticate hospital mortality. Further studies are needed to validate this finding.

### Supplementary Information

Supplementary Information.

## Data Availability

Data will be made available by Dr. Peter C. Hou upon reasonable request.

## Abbreviations

ARDS: Acute respiratory distress syndrome
RoCI: Registry of critical illness
ICU: Intensive care unit
ELISA: Enzyme-linked immunosorbent assay
CV: Coefficient of variability
RAS: Renin-angiotensin system
ACE: Angiotensin converting enzyme
Ang II: Angiotensin II
APACHE: Acute physiology and chronic health evaluation
SOFA: Sequential organ failure assessment
BMI: Body mass index
COPD: Chronic obstructive pulmonary disease
D0 Renin: Day 0 renin
D3 Renin: Day 3 renin
SD: Standard deviation
OR: Odds ratio
REF: Reference
No.: Number
CI: Confidence interval
ATHOS-3: Angiotensin II for the Treatment of Vasodilatory Shock
PRA: Plasma renin activity
