# Supplementary material for: Plasma renin as a novel prognostic biomarker of sepsis-associated acute respiratory distress syndrome
Source: Sci Rep. 2024 Mar 20;14:6667. doi: 10.1038/s41598-024-56994-3 (PMC10954703; doi:10.1038/s41598-024-56994-3)
Supplement: Supplementary file 1 — Supplementary Information. [file 41598_2024_56994_MOESM1_ESM.docx]

**APPENDIX**

**Appendix Table 1** ΔRenin Definition

| Categories | Day 0 Renin | Day 3 Renin | Number of Patients in Category |
| --- | --- | --- | --- |
| 0 | Low_Normal | Low_Normal | 15 |
| 1 | Elevated | Low_Normal | 5 |
| 2 | Low_Normal | Elevated | 4 |
| 3 | Elevated | Elevated | 8 |

**Appendix Table 2a** Expanded Baseline Characteristics of Survivors

**
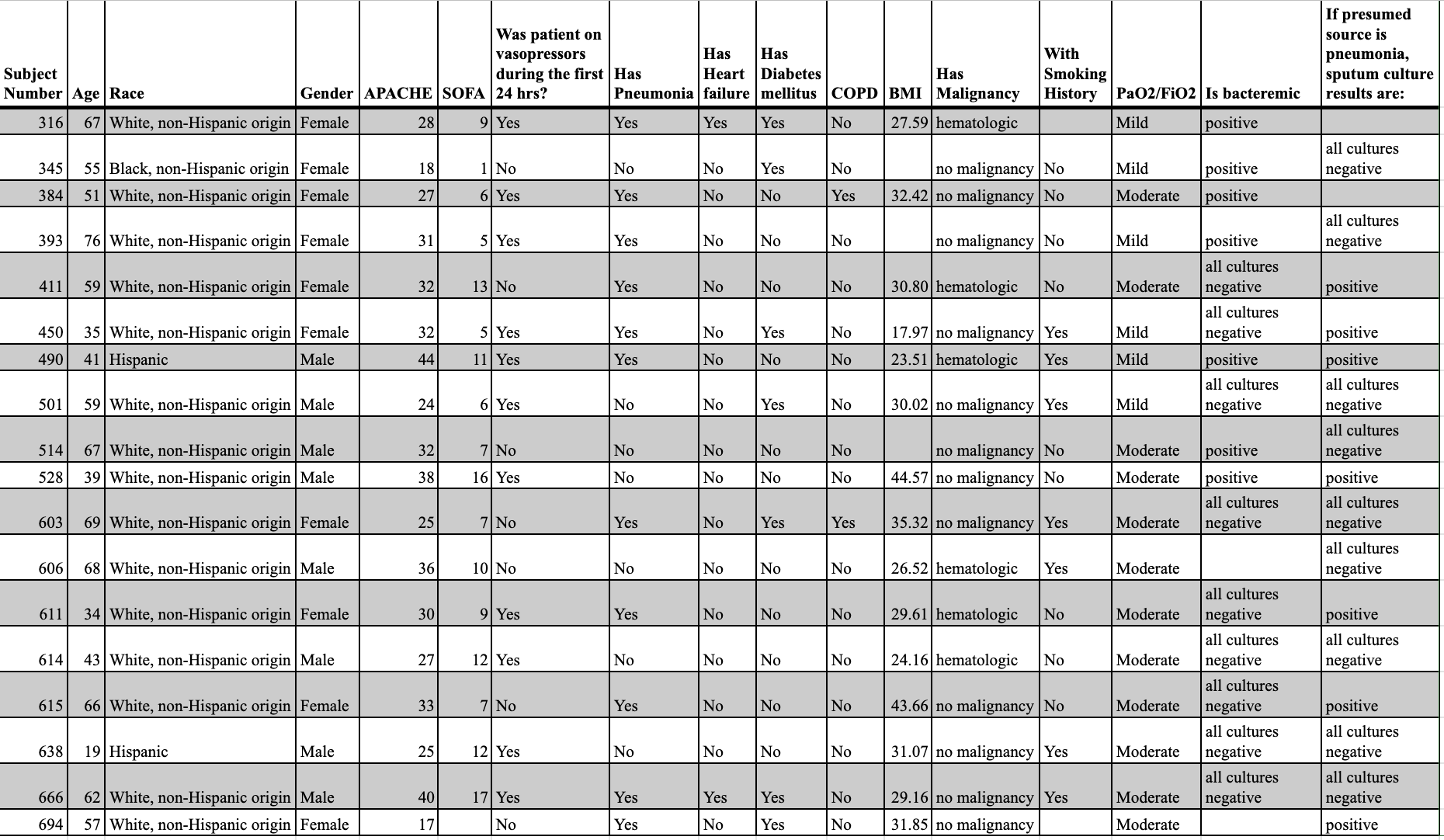
**

**Appendix Table 2b** Expanded Baseline Characteristics of Nonsurvivors

**
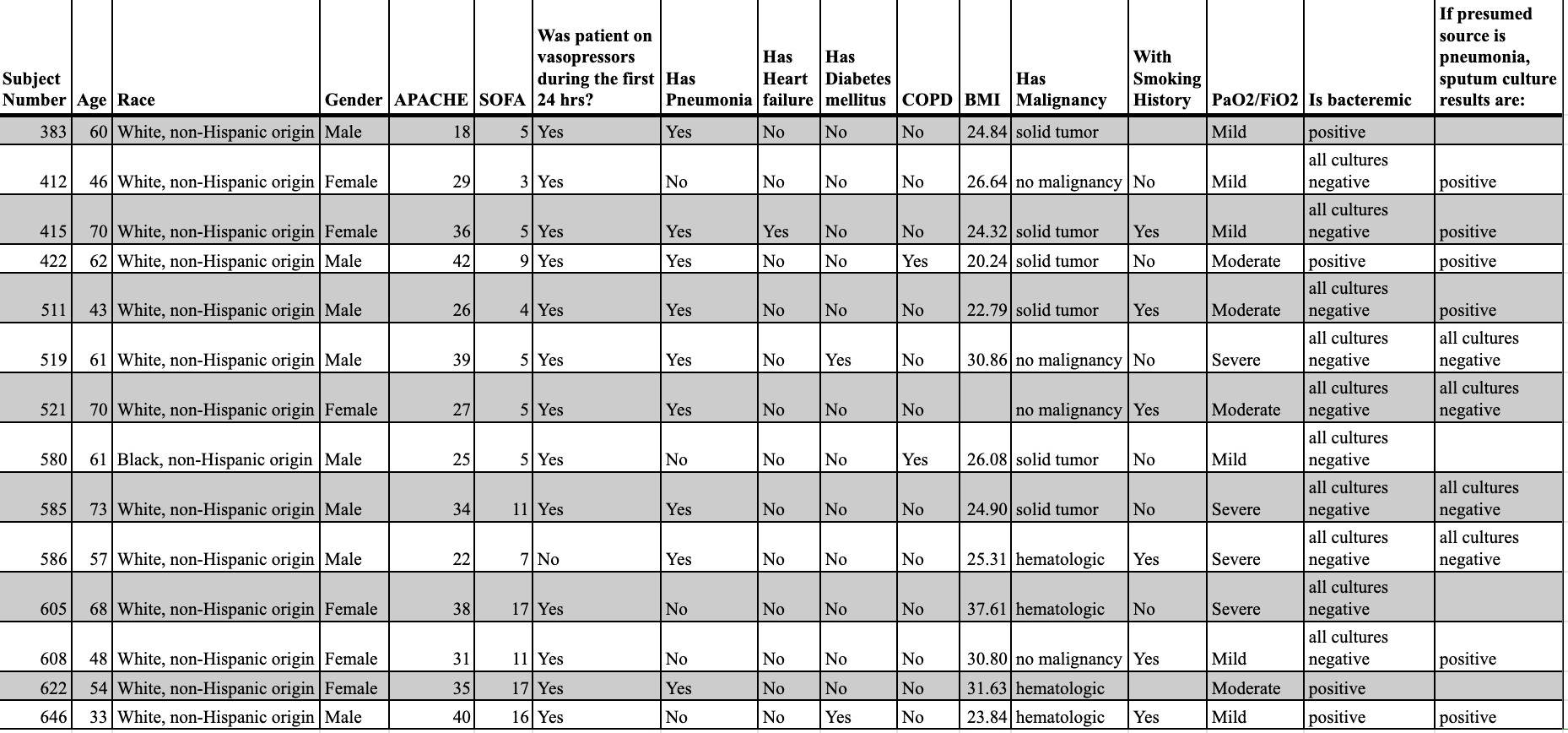
**

**Appendix Table 3a** Diagnostic Test Accuracy of Binary Day 0 Renin Level on 30-Day Hospital Mortality

| Sensitivity | 53.85% | 25.13% to 80.78% |
| --- | --- | --- |
| Specificity | 68.42% | 43.45% to 87.42% |
| Positive Likelihood Ratio | 1.71 | 0.74 to 3.92 |
| Negative Likelihood Ratio | 0.67 | 0.35 to 1.31 |

**Appendix Table 3b** Diagnostic Test Accuracy of Binary Day 3 Renin Level on 30-Day Hospital Mortality

| Sensitivity | 61.54% | 31.58% to 86.14% |
| --- | --- | --- |
| Specificity | 78.95% | 54.43% to 93.95% |
| Positive Likelihood Ratio | 2.92 | 1.11 to 7.72 |
| Negative Likelihood Ratio | 0.49 | 0.24 to 1.01 |

**Appendix Table 4** Sensitivity Analysis of Renin as a Continuous Variable

|  | Univariate | | Multivariate* | | |
| --- | --- | --- | --- | --- | --- |
|  | Mean Differences (95% CIs) | P-Values | Mean Differences (95% CIs) | P-Values | |
| Day 0 Renin | 0.0016 (-0.001 to 0.0041) | 0.241 | 0.0009 (-0.0015 to 0.0033) | | 0.452 |
| Day 3 Renin | 0.0018 (0.0004 to 0.0033) | 0.024 | 0.0014 (0 to 0.0028) | | 0.062 |
| ΔRenin | 0.0011 (-0.0007 to 0.0022) | 0.166 | 0.0009 (-0.0007 to 0.002) | | 0.208 |

*Adjusted for Vasopressors

**Appendix Figure 1** Survival Curve Stratified by Day 3 Renin Level

*Unadjusted survival curves are presented

**Appendix Figure 2** Survival Curve Stratified by Day 0 Renin Level

*Unadjusted survival curves are presented

**Appendix Figure 3** Receiver Operating Characteristic Curve for a Model Using the Continuous D3 Value to Predict 30-day In-Hospital Mortality
